# The perceptions of preclinical and clinical dental students to altered smile aesthetics

**DOI:** 10.1038/s41405-020-00045-2

**Published:** 2020-09-14

**Authors:** Maha Aljefri, Julie Williams

**Affiliations:** University of Bristol Dental Hospital, Lower Maudlin Street, Bristol, BS1 2LY UK

**Keywords:** Orthodontics, Dental education

## Abstract

**Introduction:**

This prospective cohort study was designed to identify which components of a smile make it more or less aesthetically acceptable to dental students.

**Aim:**

To investigate whether students at different stages of their undergraduate dental education held similar views on smile aesthetics. Additionally, to see whether students from the same ethnicity were more likely to have similar perceptions of smile aesthetics than students from different backgrounds.

**Methodology:**

Dental students in either Year 1 (preclinical) or Year 5 (clinical) of their studies at the University of Bristol were asked to complete a questionnaire. Students were asked to rank 12 photographic images in order from most aesthetically pleasing (1) to least pleasing (12). The 12 images included one ‘ideal’ smile and 11 digitally altered images of the same “ideal” smile.

**Results:**

A total of 123 questionnaires were completed. Clinical students were more likely to rank the ‘ideal smile’ as more aesthetically pleasing and identify it as the “best” smile from the set of images. Preclinical students considered retroclined incisors to be significantly less pleasing than clinical year students, whilst clinical year students found a midline diastema significantly less pleasing than preclinical students.

**Conclusions:**

Dental students at different stages of their undergraduate dental education have different perceptions of smile aesthetics. There was no evidence that the perception of dental attractiveness was affected by students’ ethnicities or location of upbringing.

## Introduction

Physical appearance plays a major role in human behaviour and social interaction amongst most cultures and age groups.^1^ Facial attractiveness is highly associated with attractiveness of the smile and the mouth is considered to be one of the most significant elements of appearance.^2^ A device designed to track human eye movement, for example, between two strangers talking to each other shows the attention of each individual is directed towards the eyes of the other.^3^ However, if one participant in the conversation has a compromised dentition, particularly a severe malocclusion, the gaze of the other participant is redirected towards the mouth. The appearance of the teeth has little effect on perceived facial attractiveness until a malocclusion is detected; the presence of a malocclusion has a negative impact on the perception of overall attractiveness.^4^

Although the concept of an aesthetically pleasing smile is a highly subjective and complex phenomenon, an “ideal” smile has been defined as:-

“A smile that has both harmonious correlation between the shape and colours of the teeth and a good proportion between lip and gum”.^5^

Both the soft tissues and the dentition contribute towards an ideal smile.^6^ Increased pressure from society and the media may be driving an increased demand for a perfect smile;^7^ although patients seek orthodontic treatment for a number of reasons^8^, the majority (93.4%) do so partially or exclusively due to aesthetic concerns.^9^

The appearance of a smile is largely subjective although the dental profession has created reliable, standardised and reproducible systems for ranking malocclusions according to treatment need that include an aesthetic component to the scale: the Dental Aesthetics Index (DAI)^10^ and the Index of Orthodontic Treatment Need (IOTN).^11^ Although other systems are available, those two systems are the most commonly cited indices within the orthodontic literature.^12^ Both indices have limitations: IOTN has separate scales for the clinical and aesthetic features of the malocclusion but is easy to use whilst the DAI combines both clinical and aesthetic features in one continuous scale but is considered to be less reproducible.^13^ In the United Kingdom (U.K.) and most European countries, IOTN is the index of choice whilst the DAI is more routinely used in Brazil, Saudi Arabia and the United States.^14^ Other indices such as the Index of Complexity Outcome and Need (ICON) and the Handicapping Labio-Lingual Deviation (HLD) are not as popular due to certain limitations; HLD does not record impacted or missing teeth and ICON has been described as an aesthetically orientated non-objective system.^15^

Several studies have been undertaken to investigate how general dentists, specialists and laypersons judge dental appearance.^16–19^ Smile interpretation and specifically how beauty is measured by laypersons has been found to be highly subjective and influenced by multiple factors such as culture, upbringing, ethnicity, age and gender.^20^ A Brazilian study of dental students compared tooth characteristics and factors affecting aesthetics such as staining, white spots and rotations of the teeth; dental students judged interdental spacing between anterior teeth (‘black triangles’) to be the most unacceptable characteristic of an aesthetic smile,^21^ but the study was limited as all the images were shown to students for a single short period of 20 s, before moving on to the next image. Moreover, the images were presented using a light projector, and although the authors ensured that the lighting was appropriate, projectors are known to enlarge photos and decrease the acuity of the image; certain features such as staining of the teeth may not therefore have been easily detected by the students which could explain why staining was not judged as aesthetically unacceptable. Students in the Faculties of art and science at a University within Saudi Arabia judged photographs of smiles which demonstrated different lip lines.^22^ Students found smiles with least incisal ‘show’ to be the least acceptable, where the upper lip covered 4 mm of the upper incisors. The image considered to be the most aesthetically acceptable showed all the upper incisors on smiling in addition to 2 mm of the upper gingivae. Interestingly, dental students did not judge smiles differently to students from other Faculties; results were similar across all university Faculties.

At the University of Valencia, Spain, Espana et al.^23^ explored the ability of dental students in different years of their degree to detect minor alterations in photographs of smile aesthetics. It was found that student capability of detecting anomalies did not improve as students progressed through their dental studies. However, only five variations of smiles were considered. This study also showed that students who had undergone orthodontic treatment themselves were equally unaware of dental anomalies within the photographs as those students who had not received orthodontic treatment.

In summary, previous studies of students’ perceptions of smile aesthetics have tended to compare different levels of one smile component (for example high versus low lip line) or compare different healthcare professionals’ or laypersons’ perceptions of dental variation. Even when dental students were included in studies, only a few dental variations were compared, and focused upon comparing qualified professionals’ opinions versus that of students.

The aim of this study was to compare two groups of dental students to see if there were differences in their perception of smile aesthetics and to see if this was associated with the students’ stage of dental study, their ethnicity or where they had grown up.

## Method

**Fig. 1:**
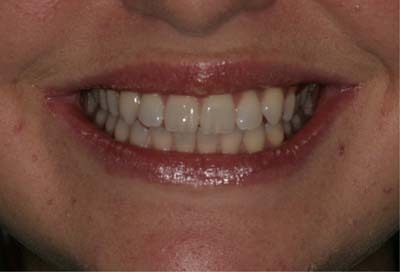
Photograph of ideal smile. The original unaltered photograph of a smiling model.

**Fig. 2:**
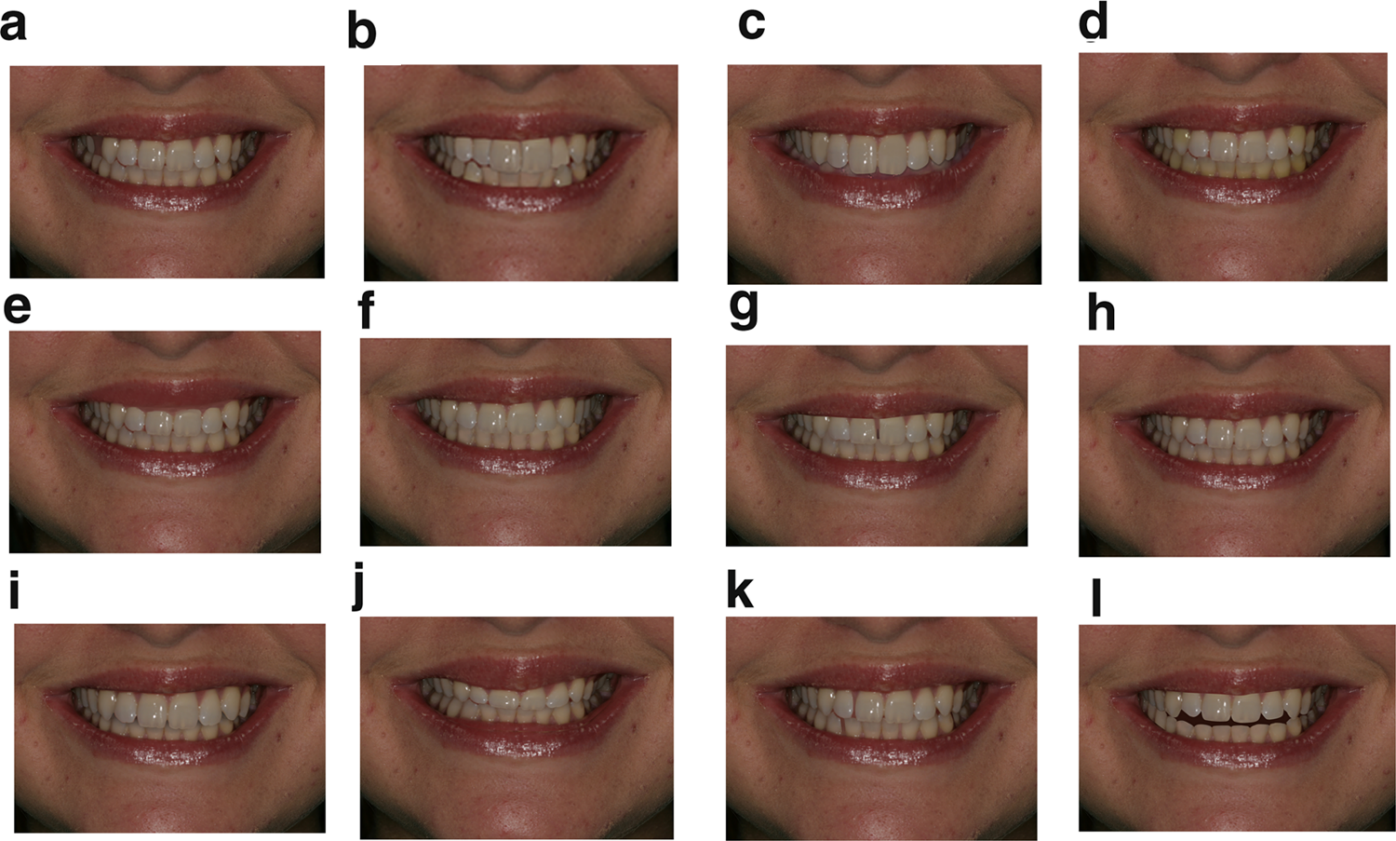
Set of 12 images including the ideal smile image. The original as well as digitally altered smiles each allocated a letter and presented to the students for ranking without their descriptions.

A suitable model was identified who presented with a Class I incisor relationship on a Class I skeletal base with Class I molar relationship, aligned teeth, healthy periodontal tissues and no anterior restorations or staining. Whether the model was male or female was random and dependent on the availability of a smile that fulfilled the criteria; the model was asked to take part and consent was obtained for the use of her photograph. The initial image of the model was captured using a digital camera (Nikon D60; New York, USA), and then cropped to exclude the nose and chin leaving an image of the lips, dentition and less than 10 mm of the surrounding skin (Fig. 1). For the purposes of the study, this smile was deemed “ideal”. A set of 12 digital images were created (Fig. 2); one image was the ideal smile and the remaining eleven were modified using Adobe^®^ Photoshop^®^ (CC, Adobe Systems, San Jose, CA, USA) to alter one feature of the smile, each image and the dental feature that had been created within the smile was identified with a letter:

**Table:** 

Image A: a missing tooth	Image B: crowded teeth
Image C: a deep overbite	Image D: discoloured teeth
Image E: low upper gingival margin	Image F: a shifted maxillary midline
Image G: a midline diastema	Image H: the original image
Image I: proclined incisors	Image J: retroclined incisors
Image K: spaced teeth	Image L: an anterior open bite (AOB)

First and fifth year undergraduate dental students at the University of Bristol were invited to complete a previously piloted questionnaire (Appendix 1) to include details of their age, ethnicity, country of birth and country of their early development, which was defined as the first 10 years of life. The students were presented with a colour sheet of photographs and asked to rank the aesthetics of the 12 digital images of the same smile, with 1 for the most aesthetically pleasing smile and 12 for the least. Participation was voluntary and anonymous. Faculty ethical approval was obtained prior to the study and the dental students consented to their data being gathered, analysed and reported for the purpose of this study. Data were collected at the beginning of the academic year, which meant that fifth year students had completed all their clinical assessments but were yet to sit their Bachelor of Dental Surgery final examinations. First year students were approached during a lecture in their first week of attending dental school, meaning they were yet to receive any dental education.

The results were manually copied onto a Microsoft Excel (2013) spreadsheet. The rankings of the 2-year groups and those raised in the U.K. or internationally were compared using the Mann–Whitney test. The three groups of different ethnicities were compared using the Kruskal–Wallis test. The *p* values were calculated and data analyses performed using IBM SPSS Statistics (Macintosh, Version 22.0. Armonk, NY).

## Results

123 questionnaires were completed, 69/72 by Year 1 (preclinical) students (95.8% response rate) and 54/66 by Year 5 (clinical) students (81.8% response rate). Questionnaires (*n* = 2) that were completed but then excluded from the study were ranked incorrectly (for example, used the same number twice) or illegible. Participants had a mean age of 20.8 years (range 18–39 years, SD 3.1). Year 1 students averaged 19.1 years (SD 2.3) and Year 5 23.0 years (SD 2.7).

99% of the students sampled were born and raised in the U.K., hence the country of birth was not included in the analysis. Out of the 121 students, only 14 spent their childhood outside the U.K., and the participants were therefore divided into two further groups, those who grew up in the U.K. and those who lived elsewhere whilst they were growing up. 89.3% of the students were of White (*n* = 67) or Asian (*n* = 34) ethnicities so the remainder (Arab, Black, Chinese or mixed) were analysed as a group (*n* = 20).

### Comparison of perception of preclinical and clinical dental students

Preclinical (Year 1) and clinical (Year 5) students had similar perceptions of the smiles that were not ideal e.g. presented with a missing tooth, crowded teeth, a deep overbite, discoloured teeth, a low upper gingival margin, an upper dental midline that was not coincident with the facial midline, proclined incisors, spaced teeth and an anterior open bite (*p* > 0.001)(Table 1).

**Table 1:** Comparing image rankings of Year 1 and Year 5 students.

	Median (Interquartile range)		*p* value	95% CI
	Year 1 (preclinical)	Year 5 (clinical)		
Image A (a missing tooth)	5.0 (3.5, 6.0)	5.0 (3.0, 7.0)	0.9	(−1.0 to 1.0)
Image B (crowded teeth)	10.0 (8.0, 11.0)	10.0 (9.0, 12.0)	0.5	(−1.0 to 0.0)
Image C (a deep overbite)	7.0 (5.0, 9.0)	7.0 (6.0, 9.0)	0.55	(−1.0 to 1.0)
Image D (discoloured teeth)	5.5 (3.0, 9.0)	4.0 (2.0, 6.0)	0.02	(0.0 to 3.0)
Image E (low upper gingival margin)	6.0 (4.0, 8.0)	6.0 (4.0, 7.0)	0.31	(0.0 to 1.0)
Image F (a shifted maxillary midline)	3.5 (2.0, 5.0)	4.0 (3.0, 6.0)	0.26	(−1.0 to 0.0)
Image G (a midline diastema)	9.0 (7.0, 10.0)	11.0 (10.0, 12.0)	*p* < 0.001	(−3.0 to −1.0)
Image H (the original image)	1.0 (1.0, 2.0)	1.0 (1.0, 1.0)	*p* < 0.001	(0.0 to 0.0)
Image I (proclined incisors)	6.0 (3.0, 8.0)	8.0 (4.5, 9.0)	0.02	(−2.0 to 0.0)
Image J (retroclined incisors)	9.0 (8.0, 11.0)	7.0 (5.0, 9.0)	*p* < 0.001	(1.0 to 3.0)
Image K (spaced teeth)	4.0 (3.0, 5.8)	5.0 (3.0, 7.0)	0.03	(−2.0 to 0.0)
Image L (an anterior open bite)	12.0 (11.0, 12.0)	11.0 (10.5, 12.0)	0.02	(0.0 to 1.0)

### Analysis of different features of the smile

**Fig. 3:**
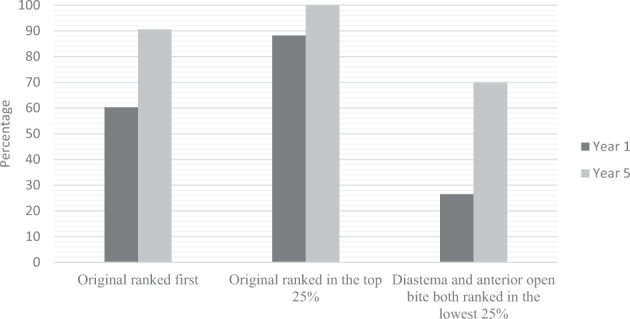
Comparison of rankings of image H, image G and image L between both year groups. A chart demonstrating percentages of pre-clinical and clinical students ranking the smile with a midline diastema and the smile with an anterior open bite collectively in their bottom three choices, as well as their ranking of the original photograph as their most favourable or one of their top three smile choices.

Clinical and preclinical students ranked different features of the smile as particularly displeasing: clinical students ranked a midline diastema to be significantly lower than preclinical students who perceived the smile with retroclined incisors as significantly less pleasing than the clinical students. All Year 5 clinical students ranked the ideal smile image amongst their top three choices and found the ideal smile image more aesthetically pleasing than Year 1 preclinical students (*p* < 0.001, CI 0.0, 0.0). Both year-groups perceived the open bite as an unfavourable feature. However, a high proportion of clinical students ranked the smiles with an anterior open bite and the midline diastema collectively in the bottom three of their rankings suggesting that they were the least favourable options of all to this year group (Fig. 3).

### Comparison of student backgrounds

There was no statistically significant difference in smile perception between students of different ethnicities nor between those who were raised in the U.K. or elsewhere (*p* > 0.001).

## Discussion

Year 5 clinical students were shown to be more likely than Year 1 preclinical students to rank the ideal smile as the most aesthetically pleasing. The study sample was taken from two cohorts of students at similar times rather than one cohort at different points within their course; it is therefore not possible to identify whether the difference in aesthetic perception was solely due to undertaking the dental programme. Dental students’ ability to identify anomalies in photographs of smiles was not previously shown to change throughout the course of their degree.^23^ It is however likely that University of Bristol Year 1 students would be similar to students in Year 5 when they started their undergraduate course 4 years ago, in terms of age, previous education and experience of dentistry. The perceptions of first year students could be similar to the opinion of laypersons, albeit individuals interested in dental studies.

A recent comparison of general dental practitioners’ and laypersons’ sensitivity to minor digital adjustments of the same smile showed that dentists have a higher sensitivity to midline diastemas of 2 and 3 mm compared with laypersons;^24^ in this study Year 5 dental students were indeed more likely than Year 1 students to rank smiles with midline diastemas lower than other smiles. It was initially surprising that the shifted maxillary dental midline smile was not identified as aesthetically displeasing by either year group as a systematic review of laypersons’ perception of smile aesthetics showed a sensitivity to 2 mm of acceptable discrepancy between the mandibular and maxillary midlines.^25^ However the manipulation of the ideal smile image was possibly not dramatic enough to create more than a 2 mm discrepancy which could explain why the midline discrepancy image was not ranked lower down the list. Kokich et al.^26^ compared laypersons, dentists and orthodontists’ ideas of dental aesthetics and found that orthodontists were more sensitive to a 1.0–1.5 mm distance between central incisors, whereas dentists and laypersons only deemed smiles less attractive when the midline diastema was 2 mm. It was therefore surprising to see that Year 5 students detected the subtle midline diastema within the image and deemed it significantly more displeasing than Year 1 students.

Zawawi et al.^22^ found that a smile showing 2 mm of the upper gingivae was ranked by University students as the most pleasing smile. Our manipulation of the original image to create a low gingival margin also showed 2 mm of upper gingivae but received a low median ranking of 4. This could be due to the interplay of other factors affecting the aesthetics of the smile, for example a smile line above the gingival margin can be obtained by having a high lip line or simply small teeth.

There was no significant difference detected between the aesthetic perception of students with different ethnicities and whether they were raised in the U.K. or elsewhere. Similar studies of aesthetics conducted in different countries produced different results to each other,^2, 22, 23^ possibly because smile perception is affected by other factors rather than just the ethnicity and upbringing of the student. Gender, age, ethnicity, upbringing as well as individual characteristics and personality traits have all been found to have an influence on smile preferences.^27^ Studying such correlations would prove to be extremely complex and would require a significantly large and diverse sample group from multiple countries and social backgrounds.

### Limitations

This study was limited by the small sample size and that information on gender was not requested from students. The original image included lipstick suggesting a female model. Since the gender of the students was not recorded, it remains unknown whether the rankings were affected by the gender of the student, the model or a combination of the two.

Each image was manipulated to include only one specific feature with no variation in the degree of the feature of the malocclusion. For example, the midline diastema could have been 1 or 6 mm wide, or the amount of crowding portrayed could have been mild, moderate or severe. This introduces the uncertainty of whether the degree of malocclusion played a role in the students’ choices.

The intra-rater reliability of the results could have been measured by inserting an image twice into the list of images. For example, if an image of crowded teeth was inserted in two different locations on the questionnaire sheet, but was not ranked in a similar manner by students, this would either suggest that students were not carefully considering their choices and ranking randomly or that the students were not sufficiently aesthetically aware to detect identical smiles. This would have been more appropriate if the students were asked to score the smiles rather than rank them in order, as it would have appeared odd to have two identical smiles in the selection for ranking.

Unlike previous studies, students were asked to rank the images and so the median and interquartile ranges were used to calculate the *p* values. If students had been asked to simply score the photographs, for example from 1 to 5, with each score being used more than once, it would have been possible to calculate the mean, which would have been a stronger method of analysis. On the other hand, by asking students to rank the images, we eliminated the possibility of one image being equally scored with another, encouraging the students to think of which image they truly preferred.

### Future research

There was a high response rate from students to take part in this study, which may be related to the simplicity of the questionnaire format or possibly that the topic was appealing to dental students.

Recommendations for further work would include a larger sample size from a number of dental schools, which may be more representative of the national dental student population. It would also be interesting to undertake a similar study internationally to compare rankings with students who are born and raised in other countries. Further studies should include gender and possibly other factors that may influence smile perception such as previous experience as a dental care professional or being the child of parents within the profession. Repeating one or two images within the study and also repeating the study at a later date would allow measurement of intra-rater reliability but would increase the burden of the research upon the participants. A longitudinal study design where students are asked to rank images at repeated time-points throughout their course may provide more information about the effect of the undergraduate degree programme upon the smile perception of dental undergraduate students, if factors other than age remained constant. Lastly, each image with a particular feature could be altered by a little or a lot giving a range of images for the same smile feature, which may affect the ranking.

## Conclusions

There is a difference in perception of smile aesthetics between preclinical and clinical dental students. Clinical students in their last year of undergraduate dental education ranked the image of the ideal smile as one of the most aesthetically pleasing smiles. A smile with a midline diastema was considered significantly less pleasing to clinical students whilst preclinical students found retroclined incisors significantly less pleasing than clinical students. The ethnicity and upbringing of students had no statistically significant effect on smile perception.

## Appendix 1

### Student questionnaire

By completing this questionnaire, you are consenting for this information to be used for the purposes of this research project. Thank you for your participation.

Please do not write your name on this sheet, to ensure your responses remain anonymous.

Please answer the following questions:

- Age: ________
- City and country of birth: ________________________
- City and country of early development (first 10 years of life): ____________________
- Ethnicity (please tick):

-    Arab or Arab British
-    Asian or Asian British
-    Black or Black British
-    Chinese or Chinese British
-    White or White British
-    Other (please specify): ____________________

- Year of BDS programme (please circle): 1 2 3 4 5

Please rank the following smile images from 1 to 12:

1 = most aesthetically pleasing smile (in your opinion)

12 = least aesthetically pleasing smile (in your opinion)
